# EMT transcription factors snail and slug directly contribute to cisplatin resistance in ovarian cancer

**DOI:** 10.1186/1471-2407-12-91

**Published:** 2012-03-19

**Authors:** Alexandria M Haslehurst, Madhuri Koti, Moyez Dharsee, Paulo Nuin, Ken Evans, Joseph Geraci, Timothy Childs, Jian Chen, Jieran Li, Johanne Weberpals, Scott Davey, Jeremy Squire, Paul C Park, Harriet Feilotter

**Affiliations:** 1 Department of Pathology and Molecular Medicine, Queen's University, 88 Stuart Street, Kingston, ON, K7L3N6, Canada; 2 Division of Gynaecologic Oncology, The Ottawa Hospital, 501 Smyth Road, Ottawa, ON, K1H8L6, Canada; 3 NCIC Clinical Trials Group, Cancer Research Institute, 10 Stuart Street, Kingston, ON, K7L3N6, Canada; 4 Ontario Cancer Biomarker Network, 101 College Street, Toronto, ON, M5G 1L7, Canada

## Abstract

**Background:**

The epithelial to mesenchymal transition (EMT) is a molecular process through which an epithelial cell undergoes transdifferentiation into a mesenchymal phenotype. The role of EMT in embryogenesis is well-characterized and increasing evidence suggests that elements of the transition may be important in other processes, including metastasis and drug resistance in various different cancers.

**Methods:**

Agilent 4 × 44 K whole human genome arrays and selected reaction monitoring mass spectrometry were used to investigate mRNA and protein expression in A2780 cisplatin sensitive and resistant cell lines. Invasion and migration were assessed using Boyden chamber assays. Gene knockdown of *snail *and *slug *was done using targeted siRNA. Clinical relevance of the EMT pathway was assessed in a cohort of primary ovarian tumours using data from Affymetrix GeneChip Human Genome U133 plus 2.0 arrays.

**Results:**

Morphological and phenotypic hallmarks of EMT were identified in the chemoresistant cells. Subsequent gene expression profiling revealed upregulation of EMT-related transcription factors including *snail, slug, twist2 *and *zeb2*. Proteomic analysis demonstrated up regulation of Snail and Slug as well as the mesenchymal marker Vimentin, and down regulation of E-cadherin, an epithelial marker. By reducing expression of *snail *and *slug*, the mesenchymal phenotype was largely reversed and cells were resensitized to cisplatin. Finally, gene expression data from primary tumours mirrored the finding that an EMT-like pathway is activated in resistant tumours relative to sensitive tumours, suggesting that the involvement of this transition may not be limited to *in vitro *drug effects.

**Conclusions:**

This work strongly suggests that genes associated with EMT may play a significant role in cisplatin resistance in ovarian cancer, therefore potentially leading to the development of predictive biomarkers of drug response or novel therapeutic strategies for overcoming drug resistance.

## Background

Of the gynecological malignancies, ovarian cancer has the highest associated mortality rate in the western world [1,2]. While relatively rare at 1 in 71 women affected in Canada [3], approximately 70-80% of patients with ovarian cancer will succumb to the disease within five years of diagnosis [4]. The high mortality rate is due, in part, to the fact that ovarian cancer is often diagnosed in advanced stage, because of a lack of measurable early symptoms and ineffective screening techniques [5,6]. Of equal importance, 20% of tumours display primary resistance to platinum compounds while the majority of initial responders will relapse, often as a result of acquired drug resistance [7,8].

Standard treatment for ovarian cancer involves tumour debulking and platinum-based chemotherapy administered intravenously or intraperitoneally [9,10]. Cisplatin, the most common first line chemotherapeutic drug, is a platinum compound that binds to and cross-links DNA [11]. During cell division cisplatin-DNA adducts block replicative machinery, inducing the DNA damage response, and eventually apoptosis [11,12]. It has been proposed that decreased cellular uptake of drug as well as increased capacity for DNA damage repair and anti-apoptotic signaling may play a role in cisplatin resistance displayed by many tumours [12-17].

Recent evidence has suggested that processes of the epithelial to mesenchymal transition (EMT) may play a role in the development of chemoresistance. EMT is a critical process in embryogenesis [18] and has been well studied in that context. It is characterized by up-regulation of extracellular matrix components, a loss of intercellular cohesion, increased rate of cellular migration and invasion, as well as increased resistance to apoptosis, and is modulated by a number of transcription factors, namely SNAI1 (Snail) and SNAI2 (Slug) [19,20]. In early embryogenesis, these cellular traits enable both the formation of the germinal layers during gastrulation by facilitating formation of the mesoderm and endoderm from cells in the primitive streak, and derivation of migratory neural crest cells from the epithelial neural plate [21]. EMT also has a significant role later in embryo development during tissue reorganization and organ modeling [22,23]. The same cellular remodeling and signaling networks appear to be active during metastasis, and may also contribute to the development of drug resistance in tumour cells [24-26]. During cancer progression, EMT appears to promote dissemination of cells from the tumour mass [27] and facilitates tissue invasion by regulating the production of matrix metalloproteases and altering cytoskeletal organization [28,29]. In models of drug resistant breast and ovarian cancers, EMT gene signatures have been found to correlate with the presence of drug resistance [30,31] and manipulation of EMT transcriptional regulators modulates resistance to chemotherapeutic drugs in lung and bladder cancers [32,33]. Additional evidence suggests that EMT may contribute to the acquisition of drug resistance by altering expression of key genes involved in cell cycle regulation, drug transport and apoptosis [34-36].

We hypothesized that the genes that regulate EMT have a role in cisplatin resistance in ovarian cancer. Using a cell line model of cisplatin-resistant ovarian cancer, we demonstrate features of a mesenchymal phenotype in the resistant cells relative to their epithelial parent cells. Additionally, we demonstrate increased capacity for migration and invasion in resistant compared to sensitive cells, characteristics that are reverted following reduction of expression of *snail *and *slug*. Using a cohort of primary human ovarian tumours, we demonstrate that EMT genes are upregulated in chemonaïve drug resistant tumours, suggesting that these genes may act as biomarkers of chemotherapy resistance. Finally, we modulate resistance to cisplatin in A2780cis cells by manipulating levels of *snail *and *slug*, suggesting that the key regulators of EMT are directly contributing to the drug resistant phenotype in ovarian cancer cells.

## Methods

### Cell lines and culture conditions

The cisplatin-sensitive A2780 ovarian adenocarcinoma cell line and its daughter line, A2780cis, were obtained from the European Collection of Cell Cultures (Salisbury, UK). Cells were cultured in RPMI with 10%FBS, 1% penicillin/streptomycin and 1% L-glutamine at 37°C in 5% CO_2_. A2780cis cells were maintained in media with 1 μM cisplatin. For all assays, cells were grown to 70-80% confluence and harvested following trypsinization. Analysis of cell morphology was done at 20× magnification using a Zeiss Axiovert 25 Phase Contrast Inverted Microscope. Digital images were captured using a Canon Power Shot G10 equipped with a Carl Zeiss 426126 lens.

### Human ovarian tumours

Consent for tumour banking was obtained and the study was approved by the Research Ethics Boards at both Kingston General Hospital and The Ottawa Hospital. Tumour samples were obtained from the Division of Gynecologic Oncology Ovarian Tissue Bank and the Ontario Tumour Bank. All tumours were chemonaïve at collection. Seventeen tumours were classified as chemosensitive (progression-free interval of greater than 18 months) and eleven as chemoresistant (progression-free interval of less than 8 months) using available follow-up clinical data. Histological assessment of samples confirmed that each sample contained > 70% tumour cells.

### Gene expression profiling and analysis

Total RNA was extracted from the cell lines and tumours using the Qiagen miRNeasy Mini kit (Toronto, Canada). RNA quality was assessed to have an RNA integrity number of at least eight using an Agilent 2100 Bioanalyzer (Mississauga, Canada). For cell lines, total RNA was labeled with Cyanine-3 dye using a Quick Amp Labeling Kit (Agilent, Mississauga, Canada) and hybridized to Agilent Whole Human Genome (4 × 44 K) Microarrays (Mississauga, Canada) for 17 hours in a rotating SciGene model 700 oven (Sunnyvale, USA). Arrays were scanned using an Agilent Technologies DNA Microarray Scanner and data were feature extracted using Feature Extraction Software 10.5.1.1 (Agilent) and statistically analyzed using default settings on GeneSpring GX 11.0.1 software (Agilent).

Expression profiles from the tumor RNA were obtained using Affymetrix GeneChip Human Genome U133 plus 2.0 arrays. Raw data were imported into GeneSpring GX 11.0.1 and analyzed. Unsupervised hierarchical clustering of the tumour samples was completed using the self-organizing maps algorithm in the GeneSpring GX 11.0.1 package.

### qRT-PCR Taqman™ arrays

Snail and Slug expression levels were analyzed using Taqman™ assays (Applied Biosystems, Streetsville, Canada, item Hs00195591_m1 and item Hs00950344_m1) and the SuperScript III First-Strand Synthesis SuperMix kit for qRT-PCR (Invitrogen, Burlington, Canada). PCR conditions were 50°C for 2 minutes, 95°C for 10 minutes, 40 cycles of 95°C for 15 seconds, 60°C for 1 minute. GAPDH was used as an internal control. As a measure of relative change in expression between the parental and resistant samples, ΔΔCt values were calculated and converted to approximate fold change values (2^-ΔΔCt^) [37].

### Cell proliferation assay

Cells were plated in 24-well plates at 5 × 10^4 ^cells/well. Cells were harvested and counted using a haemocytometer after 24, 48 and 72 hours. Average cell counts were used to produce growth curves, from which cell doubling time was calculated.

### Wound healing assay

Cell migration was assessed using wound-healing assays. Cells were grown in a confluent monolayer in a 60 mm plate. A wound was inflicted in the cell layer by scratching the plate with a sterile pipette tip. Plates were rinsed gently with media twice prior to incubation to remove non-adherent cells. Digital images of the wound were obtained at times 0 hours, 12 hours and 36 hours at 10× magnification. Effects of proliferation were controlled for by using a reduced serum medium (3%FBS) and monitored via cell count.

### Boyden chamber migration and invasion assays

Cells were serum starved for 24 hours prior to use. Media with 10% FBS was added to the wells of a 24-well plate. BD Falcon™ Cell Culture Inserts (BD Biosciences, Mississauga, Canada) were placed in each well. 2 × 10^3 ^cells in serum-free media were added to the interior of each insert. Plates were incubated for 24 hours at 37°C in 5% CO_2_, and media removed from the insert, which was then washed with PBS. Insert membranes were fixed with cold methanol for 10 minutes, stained with 0.5% Crystal Violet in 25% methanol for 10 mins and rinsed with water to remove excess dye. Membranes were removed from the insert, placed under a microscope and the number of cells that migrated through the porous membrane was counted.

Invasion assays were done as described above using BD BioCoat™ Matrigel™ invasion chambers (BD Biosciences, Mississauga, Canada).

### MTT assays

5 × 10^3 ^cells/well were seeded in 96-well plates in 100 ul medium with 10 μM cisplatin and without phenol red and left to incubate for 48 hours at 37°C and 5% CO_2 _. After 48 hours, 10 μl MTT (3-(4,5-Dimethylthiazol-2-yl)-2,5-diphenyltetrazolium bromide) (Sigma-Aldrich, Oakville, Canada) was added to each well and cells were left for 4 hours. After incubation, 150 μl MTT solvent (0.1 N HCl in anhydrous isopropanol) was added to each well and mixed thoroughly by pipetting until all formazan crystals were dissolved. Colourimetric change was measured at 570 nm and background absorbance at 690 nm. Final values were obtained by subtracting OD690 nm from OD570 nm. MTT assays for siRNA optimization were done without adding cisplatin and the initial seeded cells were only incubated for 24 hours prior to MTT addition.

### Gene knockdown

Pre-designed siRNA oligos for *snail *(cat# SASI_Hs01_00039785, duplex sequences: 5'GCCUUCAACUGCAAAUACU and 5'AGUAUUUGCAGUUGAAGGC) and *slug *(cat# SASI_Hs01_00159363, duplex sequences: 5'GCAUUUGCAGACAGGUCAA and 5'UUGACCUGUCUGCAAAUGC) were purchased from Sigma-Aldrich (Oakville, Canada).

Optimization of gene knockdown was done using AllStars Hs Cell Death Control siRNA (Qiagen, Toronto, Canada), a mix of siRNAs that target genes essential for cell survival. 5 × 10^4 ^cells/well were plated in 24-well plates and incubated overnight. After 24 hours cells were transfected with 7.5 ng, 19 ng, 37.5 ng or 75 ng of AllStars Hs Cell Death Control siRNA and 1.5 μl, 3 μl or 7.5 μl of HiPerFect Transfection Reagent (Qiagen, Toronto, Canada). Cells were incubated for 72 hours and cell death measured using MTT assays. Maximum cell death was achieved using 19 ng siRNA with 3 μl of transfection reagent. Optimal time points were then established using siRNA targeted against Snail and Slug. 24, 48 and 72 hours after transfection the level of *snail *and *slug *transcript was determined by qRT-PCR TaqMan assay. Optimal knockdown of both genes was seen 72 hours post transfection. Efficiency of transfection in the A2780 and A2780cis cells was determined to be 87% and 84%, respectively.

For knockdown experiments, 5 × 10^4 ^cells/well were plated in 24-well plates and incubated overnight. 19 ng of siRNA and 3 μl of HiPerFect transfection reagent were diluted in 100 μl serum-free RPMI and were incubated for 10 minutes at room temperature. Transfection complexes were then added drop-wise onto the cells. Cells were incubated for 72 hours at 37°C and 5% CO2. Media was changed as necessary. Transfection of A2780cis cells with AllStars Negative Control (Qiagen), an siRNA sequence with no homology to any known mammalian gene, was used as a control for this experiment. Cells with reduced expression were designated as follows: A2780cisSN - *snail *knockdown, A2780cisSL - *slug *knockdown, A2780cisSN/SL - both *snail *and *slug *knocked down.

### Statistical analysis

Each assay was performed in triplicate. Data are expressed as the mean ± standard deviation (SD). Statistical significance of all data was evaluated using the Student's *t*-test, p < 0.05.

### Sample preparation for proteomic analysis

Samples were lysed in RIPA buffer containing Halt Protease and Phosphatase Inhibitor Cocktail (PIERCE, IL, USA). Cells were sonicated and lysates incubated at 4°C for 30 minutes with shaking. Supernatants were separated by centrifugation for 15 minutes at 4°C. Protein concentration was measured with a Bio-Rad DC Protein Assay kit and 30 μg of protein was used for tryptic digestion. Each aliquot was dried, dissolved in 0.5 M triethylammonium bicarbonate (TEAB), reduced by adding 2 μl of tris-(2-carboxyethyl)-phosphine (TCEP), incubated at room temperature for 1 hour, alykalted using 1 μl of methyl methane thiosulfonate (MMTS) and incubated at room temperature for 1 hour in the dark. 2 μg trypsin was added for overnight digestion at 37°C. The tryptic digest was desalted by using a C18 spin column (PIERCE, USA), dried under vaccum and resuspended in 0.1% formic acid for SRM analysis.

### LC/SRM-MS analysis

The LC/SRM-MS analytical system consisted of an Eksigent nanoflow HPLC (AB Sciex, USA) coupled to a 5500 QTRAP^® ^hybrid triple quadrupole/linear ion trap mass spectrometer (AB SCIEX, USA). 1 μg of digested protein was loaded onto a trap column (0.3 mm I.D, 5 mm L), packed with 5 μm Zorbax SB-C18, 300 Å pore (Agilent, USA). Peptides were separated on a 75 μm I.D., 15 cm long nanoflow column with 15 μm spray tip (New Objectives, USA). A linear gradient profile was employed starting from 5% solvent B to 40% B in 60 minutes (solvent A was 2% acetonitrile in water with 0.1% formic acid; solvent B was 2% water in acetonitrile with 0.1% formic acid). The ion spray voltage was set at 2300 V, and source temperature at 160°C. The declustering voltage was 100 V, and collision energy value was selected for each transition as generated by MultiQuant^® ^software (AB Sciex, USA). To ensure sensitive and reliable quantification, tryptic peptides and SRM transitions were generated by MRMPilot software (AB SCIEX, USA) based on common chemical rules of peptide fragmentation. The specificity of each peptide was verified using BLAST alignment against the NCBI-NR human protein database. Raw SRM-MS data was preprocessed using MultiQuant 2.0.2 software (AB SCIEX). A 2-point Gaussian smoothing window was applied to all transition peaks. Peak area for each transition was averaged over three replicate experiments per resistant and sensitive cell line sample; fold-change was calculated from the ratio of the average peak area in resistant cells to that in sensitive cells. A transition was discarded if peak area coefficient of variation (CV) across replicates was greater than 0.2 or peak area in any replicate was below the 25th percentile. Peptides with at least 2 transitions satisfying these constraints were conserved.

## Results

### Cisplatin resistance in A2780cis correlates with changes in cellular morphology consistent with EMT signaling

**Figure 1:**
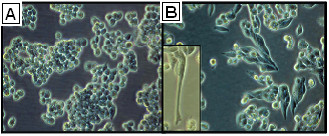
**Morphology of A2780 and A2780cis cells**. Cells visualized under 20× magnification. Drug sensitive cells (**a**) have round morphology and grow in tight clusters with substantial cellular cohesion. Comparatively, the drug resistant cells (**b**) have a more fibroblastic appearance and demonstrate reduced intercellular contacts. Additionally, drug resistant cells extend pseudopodia (inset).

We examined the morphological characteristics of the cell lines during exponential growth. Parental A2780 cells formed cohesive clusters with round cellular morphology in vitro (Figure 1a), consistent with an epithelial phenotype. In contrast, A2780cis cells, grown in the presence of 1 μM cisplatin, display a spindle-like morphology and formed dyscohesive sheets (Figure 1b). Additionally, the resistant cells exhibit the formation of pseudopodia (Figure 1b, inset), not seen in the parental cells.

### Whole transcriptome profiling and LC/SRM-MS analysis identify components of EMT signaling networks in A2780cis relative to A2780

**Figure 2:**
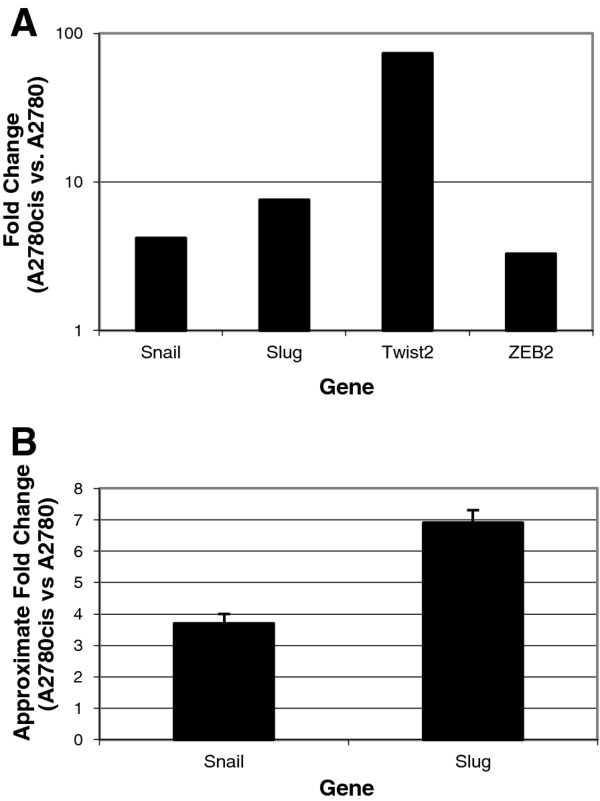
**Upregulation of genes associated with EMT in resistant cells**. Genes known to 'regulate EMT were shown to be upregulated in the A2780cis cells compared to the A2780 cells. Data from the gene expression microarrays (**a**) and technical validation of *snail *and *slug *expression levels using qRT-PCR TaqMan assay (**b**) demonstrate that upregulation. Approximate fold change calculated as ΔΔCt for *snail *is 3.7 ± 0.3 (p-value = 0.0008) and for *slug *is 6.9 ± 0.4 (p-value = 0.001).

To confirm the involvement of EMT elements in the development of cisplatin resistance, whole transcriptome microarrays (Agilent, Mississauga, Canada) were used to compare gene expression between A2780 and A2780cis cells during exponential growth. In repeated assays, EMT pathway elements, including *snail, slug, twist2 *and *zeb2*, were over-expressed in the cisplatin-resistant cell line relative to the parental line (Figure 2a). Technical validation by qRT-PCR using Taqman assays confirmed relative overexpression of *snail *by 3.7 ± 0.3 (SD) fold and *slug *by 6.9 ± 0.4 (SD) fold, in the drug-resistant cells compared to the drug-sensitive cells (Figure 2b). Protein analysis by liquid chromatography/selected reaction monitoring mass spectrometry (LC/SRM-MS) confirmed upregulation of Snail, Slug and vimentin, as well as downregulation of epithelial marker E-cadherin in the drug resistant cells relative to the drug sensitive cells (Table 1).

**Table 1:** Targeted protein analysis of A2780 and A2780cis cells.

Protein	Peptide	Fragment Change	Fold	Mean Peak Area		CV	
				
				Sensitive	Resistant	Sensitive	Resistant
E- cadherin		b8	-1.18	5.82E+04	3.22E+04	0.01	0.13
	GPFPKNLVQJK	b9	-1.55	1.57E+04	1.01E+06	0.07	0.08
		b10	-1.63	3.33E+04	2.05E+04	0.03	0.19
	
		y8	-4.48	2.10E+04	5.02E+03	0.09	0.10
	VFYSITGQADTPPVGVFIIER	y9	-4.16	8.25E+04	1.99E+04	0.02	0.12
		y10	-4.01	8.45E+04	2.11E+04	0.07	0.11

		y6	1.15	4.78E+06	2.52E+06	0.01	0.01
	EYQDLLNVK	y7	1.14	2.19E+06	2.50E+06	0.02	0.01
Vimentin		y8	1.11	1.27E+05	1.14E+05	0.02	0.05
	
		y6	1.01	4.58E+06	4.62E+06	0.02	0.03
	ILLAELEQLK	y7	1.08	7.34E+06	7.94E+06	0.04	0.05
		y8	1.10	1.11E+07	1.22E+07	0.04	0.04
		y9	1.08	1.35E+06	1.47E+06	0.04	0.04

		b3	1.25	6.00E+04	7.50E+04	0.14	0.03
Snail	SFLVR	b4	1.21	7.38E+04	8.89E+04	0.03	0.07
		y3	1.29	2.14E+04	2.76E+04	0.16	0.04

		b3	1.10	1.92E+04	2.10E+04	0.18	0.14
Slug	HFNASK	b5	1.27	2.40E+05	3.06E+05	0.06	0.16
	
		b10	1.05	9.96E+04	1.04E+05	0.13	0.07
	VSPPPPSDTSSK	b11	1.19	4.47E+04	5.34E+04	0.07	0.05
		y9	1.24	4.00E+04	4.96E+04	0.07	0.06

Summary of SRM-MS quantification results for E-Cadherin, Vimentin, Snail, and Slug. Mean peak area and coefficient of variation (CV) for each peptide fragment ion were derived from peak areas measured in three replicate assays of each of the sensitive and resistant cell line samples. Transitions with CV > 0.20 in either sample are not shown

### Cisplatin resistant cells display increased potential for migration and invasion

**Figure 3:**
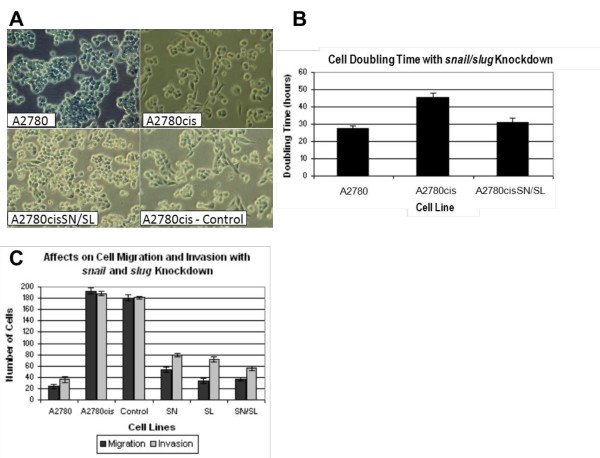
**Knockdown of *snail *and *slug *reverses EMT phenotype**. *Snail *and *slug *knockdown resulted in an epithelial morphology (**a**) and reduced the doubling time of the A2780cisSN/SL cells (p-value = 0.017) compared to the A2780cis cells (**b**). Additionally, knockdown of *snail *and *slug *resulted in a reduction in migration from 194.0 ± 7.0 cells to 33.67 ± 3.1 cells migrating through the membrane (p-value = 0.002) as well as reducing invasion rate from 188.3 ± 4.04 cells to 56 ± 4.58 invading the matrix (p-value = 0.004), in each case, making the A2780cisSN/SL knockdown cells more similar to the A2780 cell line, having migration and invasion rates of 23.7 ± 5.1 and 36.3 ± 5.03, respectively (**c**). The control group, A2780cis cells transfected with a scrambled siRNA sequence, show no statistically significant changes in morphology, doubling time, migration or invasion.

Migratory capacity was measured using Boyden chamber assays. In three independent experiments, 23.7 ± 5.1 drug sensitive cells and 194.0 ± 7.0 drug-resistant cells migrated through the membrane after 24 hours (p-value = 0.003) (Figure 3c). Resistant cells also showed a five-fold increased invasive capability relative to sensitive cells using a Matrigel-coated Boyden chamber assay. After 24 hours, an average of 36.3 ± 5.03 sensitive cells and 188.3 ± 4.04 resistant cells invaded the matrix (p-value = 0.003) (Figure 3c).

### Knockdown of snail and slug reverses the EMT phenotype and reduces cellular resistance to cisplatin

**Figure 4:**
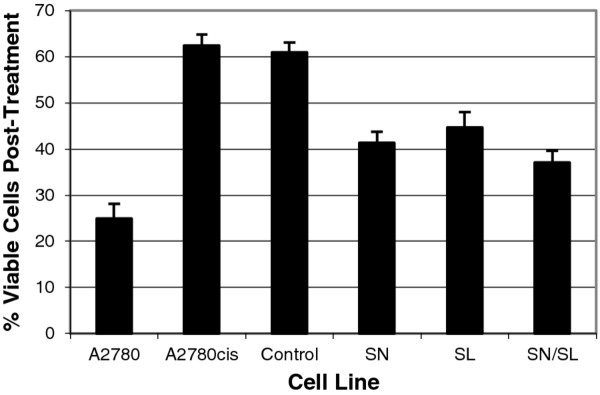
**Cisplatin sensitivity with *snail *and *slug *knockdown**. Cells with *snail *and *slug *knockdowns were grown in 10 μM cisplatin for 48 hours and cell survival was determined by MTT assay. Approximately 75 ± 3.3% of the drug sensitive cells died after treatment compared to 37 ± 2.4% of the drug resistant cells. With *snail *and *slug *knockdown, cell death was significantly increased to 62 ± 2.5% (p-value = 0.005).

SiRNA was used to reduce the levels of *snail *and *slug *transcript in the A2780cis cells to those of the parent A2780 cell line (A2780cisSN - *snail *knockdown, A2780cisSL - *slug *knockdown, A2780cisSN/SL - both *snail *and *slug *knocked down). Knockdown of these genes was confirmed by qRT-PCR (results not shown) and resulted in reversion of cell morphology of the resistant cells to that of the parent cell line (Figure 3a). Additionally, the knockdowns resulted in a reduction of the doubling time of the resistant cells from 45.7 ± 2.4 hours to 31.1 ± 2.4 hours (p-value = 0.017), similar to the 27.3 ± 1.6 hour doubling time of the drug-sensitive A2780 cells (p-value = 0.15) (Figure 3b). Migratory capacity of the transfected cells was reduced from 194.0 ± 7.0 (untransfected controls) to 33.67 ± 3.1 cells migrating through the membrane (p-value = 0.002) (Figure 3c). Invasive capability was reduced from 188.3 ± 4.04 (untransfected controls) to 56 ± 4.58 cells invading the matrix (p-value = 0.004) (Figure 3c). Finally, we showed increased cellular sensitivity to cisplatin in transfected cells relative to controls (Figure 4). Following 48 hours of growth in 10 μM cisplatin, transfected cells displayed 62 ± 2.5% cell death compared to controls at 37 ± 2.4% (p-value = 0.005). As a comparator, parental drug-sensitive cells displayed 75 ± 3.3% cell death.

### Drug resistant human ovarian tumours can be differentiated from drug sensitive ovarian tumours using a biomarker panel of EMT-related genes

**Figure 5:**
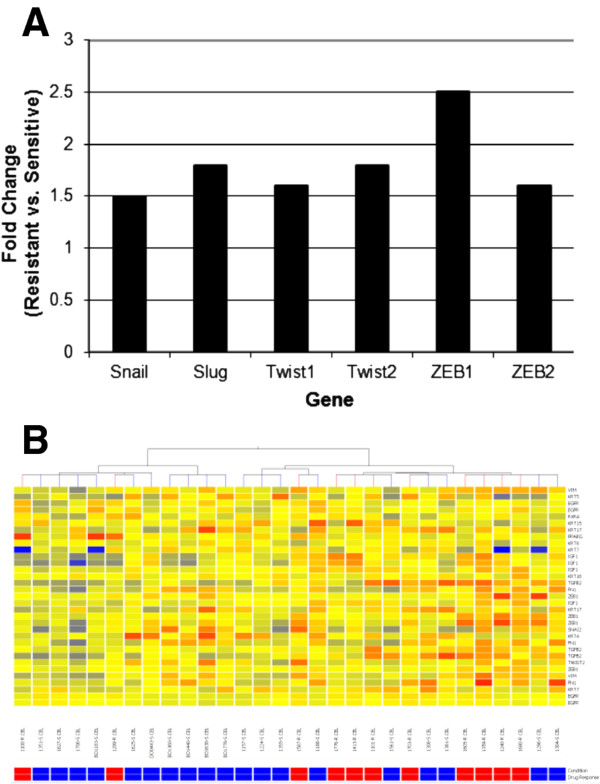
**EMT gene signatures in drug resistant ovarian tumours**. EMT gene signatures are present in primary drug resistant ovarian tumours. (**a**) Gene expression arrays identify increased expression of genes integral to EMT in the resistant tumours relative to the sensitive tumours. (**b**) Unsupervised hierarchical clustering based on a panel of genes related to EMT supports differentiation between drug sensitive (blue) and drug resistant (red) tumours.

Human ovarian tumour samples were classified as chemosensitive (progression-free interval of greater than 18 months) or chemoresistant (progression-free interval of less than 6 months) based on available clinical data (Additional file 1: Table S1). Gene expression data from 17 sensitive and 11 resistant tumours were mined to investigate the relative expression levels of EMT-related genes. In addition to *snail, slug, twist2 *and *zeb2*, the resistant tumour samples also showed increased expression of *twist1 *and *zeb1*, two other important regulators of EMT (Figure 5a). We used published information about EMT to derive a list of 17 additional genes with a role directly upstream or downstream of Snail and Slug (Table 2). Unsupervised hierarchical clustering using expression data from those genes allowed reasonable separation between the drug sensitive and drug resistant ovarian tumours (Figure 5b).

**Table 2:** List of genes used for unsupervised hierarchical clustering of primary tumours.

VIM	Vimentin
EGPR	Epidermal growth factor recetor
PPARG	Peroxisome proliferator-activated receptor gamma
IGF1	Insulin-like growth factor 1
TGFB2	Transforming growth factor beta 2
FN1	Fibronectin
ZEB1	Zinc finger E-box-binding homeobox 1
SNAI2	Slug (Zinc finger protein)
TWIST2	Twist-related protein 2
RXRA	Retinoid X receptor alpha
KRT5	Keratin5
KRT15	Keratin15
KRT17	Keratin17
KRT18	Keratin18
KRT7	Keratin7
KRT16	Keratin16
KRT4	Keratin4

Genes used to perform unsupervised hierarchical clustering on the primary ovarian tumours, chosen based on their documented involvement in EMT in recent literature

## Discussion

Ovarian cancer exhibits a high rate of platinum sensitivity in the first-line setting, but resistance frequently develops in recurrent disease [38]. As such, understanding the signaling networks that regulate chemoresistance is critical for successful treatment. This can be said of any cancer commonly treated with cisplatin such as small cell lung cancer, head and neck cancer, colorectal and hepatocelluar cancers.

While EMT has been widely studied for its role in early development and, more recently, cancer metastasis, it may also contribute to a cellular ability to evade the effects of platinum-based therapies. EMT results in the transformation of a differentiated epithelial cell to a mesenchymal cell with stem-like properties, and is characterized by loss of cell-to-cell adhesion, specifically through the dismantling of adherens, tight and gap junctions, as well as loss of cell polarity and increased motility [39]. In embryogenesis, EMT functions by promoting migration of mesenchymal cells, first, during gastrulation and neural crest cell migration, then later during tissue remodeling and organogenesis, ultimately contributing to the development of differentiated tissues with specific phenotypes [18]. In cancer progression, EMT appears to be at least partially responsible for the invasive nature of tumour cells and facilitates metastasis by converting a non-motile cancerous epithelial cell into a motile mesenchymal cell capable of disseminating from the tumour mass and entering the circulatory or lymphatic system [40]. It is widely believed that embryogenesis and cancer metastasis represent two facets of EMT, the former, a self-contained process that functions to generate diverse cell types and tissues, while the latter is affected by oncogenes and tumour suppressor genes resulting in invasion and metastatic spread through the circulatory system [41,42].

It is likely that EMT in drug resistance may rely on many of the same transcription factors that function in embryogenesis and metastasis-related EMT, although details of their regulation are largely unknown. Studies from embryology and metastasis provide evidence to suggest that these cellular changes provide a survival advantage to cells under chemotoxic stress, for example, by down-regulating pro-apoptotic factors such as caspases and *Dap1*, or up-regulating anti-apoptotic factors *Dad1 *and *Bcl2 *[43,44]. Additionally, oxaliplatin-resistant colorectal cancer cells have been shown to display many of the hallmarks of EMT, including increased migration and invasion as well as a spindle-cell shape, loss of polarity and formation of pseudopodia [45]. Increased expression of *zeb1 *and decreased expression of *E-cadherin *have been associated with drug resistant pancreatic cell lines and reduction of *zeb1 *expression has been implicated in increased drug sensitivity [46]. In ovarian cancer cell lines, upregulation of *snail *and *slug *has been correlated with resistance to radiation and paclitaxel and shown to directly participate in p53-mediated pro-survival signaling [47]. Therefore the idea that the EMT genes play a significant role in cisplatin resistance in ovarian cancer is supported by previous evidence, and our own demonstration of a direct impact of these genes on platinum resistance.

Through the manipulation of key EMT signaling molecules, we have been able to show that we can re-sensitize drug resistant cells to the effects of cisplatin, approaching wild-type sensitivity after 72 hours in culture. Our findings suggest that genes known to regulate EMT directly contribute to cisplatin resistance in this ovarian cancer model. We do recognize that the A2780 cell line may not provide a faithful model of serous ovarian carcinoma, given that lineage fidelity for this line may not be maintained in long term culture. However, we conclude that the translation of our cell line derived results to a series of primary ovarian carcinomas provides evidence for the effectiveness of this model in this instance. We have demonstrated that a panel of EMT-related genes provides a reasonable model of classifying primary ovarian tumours according to their chemoresistance status. While we recognize that optimization of the biomarker panel would be required before suggesting this could provide a clinical benefit, this initial gene list identifies key differences in the underlying molecular circuitry of drug sensitive and resistant ovarian tumours. We have not determined whether this signature derives entirely from tumour cells, given that we did not enrich for tumour cells in our experiments; however, the fact that all of the primary tumours showed at least 70% tumour cells by histology would support the idea that the tumour likely contributes substantially to the signature we noted. To our knowledge, this is the first study in primary tumours demonstrating an EMT gene signature that can be used to differentiate between chemosensitive and chemoresistant human ovarian tumours and suggests that our overall findings may provide important clues about chemotherapy resistance in ovarian cancer.

## Conclusions

In summary, we have demonstrated, through the use of an ovarian cancer cell line, A2780, and its cisplatin-resistant daughter line, A2780cis, that the genes that regulate the epithelial to mesenchymal transition directly contribute to cisplatin-resistance in ovarian cancer. We have shown that when *snail *and *slug*, two key regulators of EMT, are knocked down in our cisplatin resistant cell line, the EMT phenotype is largely reversed and drug sensitivity is restored. Additionally, we demonstrate that this gene signature is present in drug resistant human ovarian tumours and that we can distinguish between drug sensitive and drug resistant ovarian tumours using the differential expression of a panel of EMT-related genes. Therefore, as it appears that EMT is connected to the drug resistant phenotype, this process and corresponding signaling network may be relevant biomarkers of drug resistance in ovarian cancer. Additionally, these molecules may represent targets for novel therapeutic strategies, used to overcome chemotherapy resistance in ovarian cancer, thereby modulating drug response in these patients and reducing the mortality rate associated with this disease.

## Abbreviations

EMT: Epithelial to mesenchymal transition; LC/SRM-MS: Liquid chromatography/selected reaction monitoring mass spectrometry

## Competing interests

The authors declare that they have no competing interests.

## Authors' contributions

AMH carried out analysis of cell line microarray data, completed *in vitro *studies including cell migration, invasion and drug sensitivity assays, as well as gene knockdown experiments, and drafted the manuscript. MK provided microarray data for the primary tumours. MD, PN and JG carried out bioinformatic analysis of microarray data. MD and PN also designed peptide sequences for SRM-MS. KE, JC and JL carried out targeted protein quantification via SRM-MS and subsequent analysis. JW carried out classification of primary tumours as drug sensitive or resistant using clinical data. TC examined the primary tumours and calculated the percent tumour cells. SD provided guidance on biological interpretations. JS and HF conceived of the study, and participated in its design. AMH, SD, JS, PCP and HF all made significant intellectual contributions to this study. All authors contributed to the editing of this manuscript. All authors read and approved the final manuscript.

## Pre-publication history

The pre-publication history for this paper can be accessed here:

http://www.biomedcentral.com/1471-2407/12/91/prepub

## Supplementary Material

Additional file 1 **Table S1 Clinical data for ovarian tumour samples**. Available clinical data for each tissue sample including; patient's age at time of diagnosis (years), tumour stage, progression-free interval (PFI, months) and drug response classification. Click here for file
